# Diagnosis of histological type of early gastric cancer by magnifying narrow‐band imaging: A multicenter prospective study

**DOI:** 10.1002/deo2.61

**Published:** 2021-09-28

**Authors:** Takashi Kanesaka, Noriya Uedo, Hisashi Doyama, Naohiro Yoshida, Takashi Nagahama, Kensei Ohtsu, Kunihisa Uchita, Koji Kojima, Tetsuya Ueo, Haruhiko Takahashi, Hiroya Ueyama, Yoichi Akazawa, Toshio Shimokawa, Kenshi Yao

**Affiliations:** ^1^ Department of Gastrointestinal Oncology Osaka International Cancer Institute Osaka Japan; ^2^ Department of Gastroenterology Ishikawa Prefectural Central Hospital Ishikawa Japan; ^3^ Department of Endoscopy Fukuoka University Chikushi Hospital Fukuoka Japan; ^4^ Department of Gastroenterology Kochi Red Cross Hospital Kochi Japan; ^5^ Department of Gastroenterology Oita Red Cross Hospital Oita Japan; ^6^ Department of Gastroenterology Juntendo University School of Medicine Tokyo Japan; ^7^ Clinical Study Support Center Wakayama Medical University Hospital Wakayama Japan

**Keywords:** diagnosis, endoscopy, gastric cancer, prospective study

## Abstract

**Objectives:**

Distinguishing undifferentiated‐type from differentiated‐type early gastric cancers (EGC) is crucial for determining the indication of endoscopic resection. We aimed to investigate the diagnostic performance of white‐light endoscopy (WLE) and magnifying narrow‐band imaging (M‐NBI) for the histological type of EGC.

**Methods:**

In this multicenter prospective study, patients with histologically proven cT1 EGC, macroscopically depressed or flat type, size ≥5 mm, and without erosion/ulcer, were recruited. The diagnostic criterion of WLE for undifferentiated‐type EGC was pale color. The M‐NBI algorithm was created based on microsurface and microvascular patterns, and lesions with absent microsurface pattern and opened‐loop microvascular patterns were diagnosed as undifferentiated‐type. The center of the lesion was defined as the evaluation point and was initially evaluated by WLE, then by M‐NBI, and a biopsy specimen was taken as a reference standard. The primary and key secondary endpoints were overall diagnostic accuracy and specificity, respectively.

**Results:**

In total, 167 lesions (122 differentiated‐type and 45 undifferentiated‐type EGCs) in 167 patients were analyzed. The overall accuracy, sensitivity, specificity, and positive likelihood ratio of WLE for undifferentiated‐type cancer were 80%, 69%, 84%, and 4.4, respectively, and those of M‐NBI were 82%, 53%, 93%, and 7.2, respectively. There was no significant difference in overall accuracy (*p* = 0.755), but specificity was significantly higher in M‐NBI (*p* = 0.041).

**Conclusions:**

The use of M‐NBI did not improve the accuracy of WLE for the diagnosis of depressed/flat undifferentiated‐type EGCs but improved the specificity. It may reduce surgical overtreatment by preventing misdiagnosis of differentiated‐type EGC as undifferentiated‐type.

## INTRODUCTION

The histological type of gastric cancer is classified into differentiated and undifferentiated types according to Nakamura's classification,^1^, ^2^ corresponding to the intestinal and diffuse types according to Lauren's classification, respectively.^3^ The indications for endoscopic resection (ER) are more restricted for the undifferentiated type than for the differentiated type.^2^, ^4^, ^5^, ^6^ Therefore, unlike other gastrointestinal cancers, distinguishing these histological types is crucial for determining the indication of ER. Forceps biopsy is currently used for diagnosis of cancer and histological type in clinical practice when a suspicious lesion is detected by gastroscopy. Because favorable long‐term outcomes relevant to gastric endoscopic submucosal dissection for each histological type have been published,^7^, ^8^ the opportunities of ER for both histological types are increasing.

Recently, the utility of magnifying narrow‐band imaging (M‐NBI) for the diagnosis of early gastric cancer (EGC) was demonstrated.^9^, ^10^, ^11^, ^12^ NBI is an image‐enhancing technology that can be combined with magnifying endoscopy to allow for clear visualization of the microsurface structure and microvascular architecture of the gastric mucosa.^9^ The superiority of M‐NBI over white‐light endoscopy (WLE) for the differential diagnosis of small depressed EGC from benign small depression has been verified in a multicenter randomized controlled trial, which demonstrated an increase in accuracy from 64.8% to 90.4% (*p* < 0.001).^10^ However, with regard to the endoscopic diagnosis of EGC histological types, the diagnostic abilities of WLE and M‐NBI to distinguish undifferentiated type from differentiated type have not been fully analyzed. Therefore, we aimed to investigate the diagnostic performance of WLE and M‐NBI for the histological type of EGC.

## METHODS

### Study design and ethical statements

This multicenter prospective study was conducted according to the Declaration of Helsinki. The study protocol was approved by the Institutional Review Board of Osaka International Cancer Institute on December 22, 2017 (No. 1712226191) and each participating institution. This trial was registered with the University Hospital Medical Information Network Clinical Trials Registry (UMIN000032151). All participants provided written informed consent for study participation. The manuscript was described following the Standards for Reporting Diagnostic Accuracy (STARD) statement.^13^

### Patients

Patients who planned to undergo ER or gastrectomy to treat cT1 (intramucosal or submucosal) gastric cancer at the participating institutions were assessed eligibility. When eligibility criteria were confirmed, the patient agreed to participate in this trial, and written informed consent was provided, the preoperative endoscopic examination was undertaken according to the trial protocol.

Inclusion criteria were histologically proven common‐type EGC,^14^ and patients aged ≥20 years. Exclusion criteria were high risk of bleeding after biopsy (e.g., coagulation abnormality and platelet dysfunction), history of gastrectomy, the lesion of macroscopically elevated type, <5 mm in size, and evidence of erosion or an ulcer in the center of the lesion. The elevated‐type lesions were excluded because our previous study indicated that elevated‐type lesions were mostly differentiated‐type with a high positive likelihood ratio.^15^ Lesions <5 mm in size were excluded because they were smaller than the opened width of the biopsy forceps. Lesions with erosion/ulcer in the center were also excluded because endoscopic findings were unevaluable. If a patient had multiple lesions, only the largest lesion was chosen for evaluation.

Status of *Helicobacter pylori* infection was defined as follows: current infection, anti‐*Helicobacter pylori* IgG antibody was ≥10 and history of successful eradication therapy was absent; non‐infected, anti‐*Helicobacter pylori* IgG antibody was <3 and history of eradication therapy was absent; past infection, others. Tumor characteristics were described according to the Japanese Classification of Gastric Carcinoma.^14^

### Diagnostic methods

Endoscopists who were board‐certified fellows of the Japan Gastroenterological Endoscopy Society or had equivalent qualifications participated in this study as examiners. The endoscopists were blinded to the previous endoscopy report of histological findings. The targeted lesion was evaluated in WLE, and then in M‐NBI according to the algorithms described below. To eliminate selection bias, the center of the lesion was defined as the evaluation point. After completion of all diagnostic procedures, at least one biopsy specimen was obtained from the evaluation point.

### Evaluation with WLE

**FIGURE 1:**
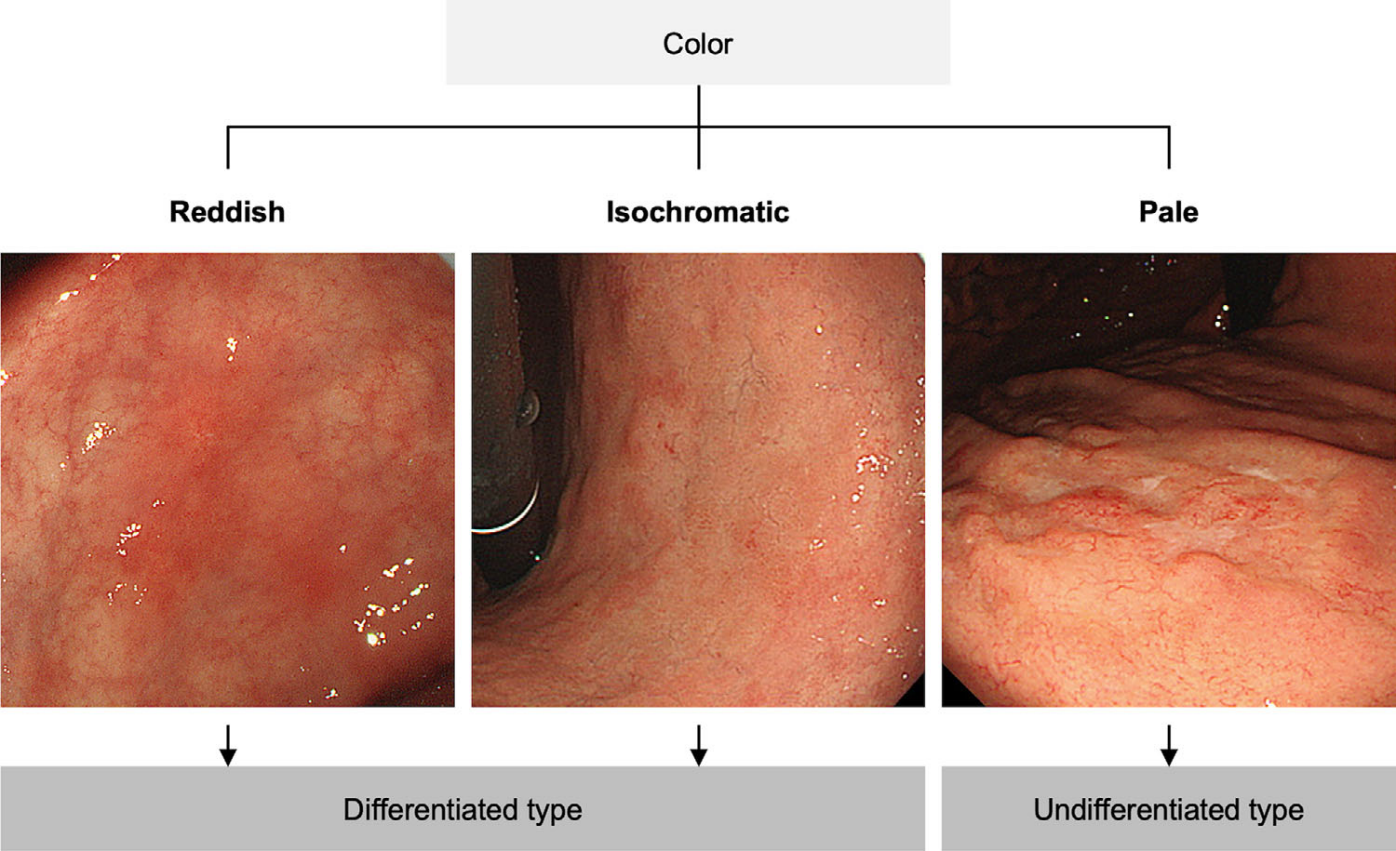
Diagnostic algorithm of white‐light endoscopy for differentiating undifferentiated‐type from differentiated‐type gastric cancer. A pale lesion is endoscopically diagnosed as an undifferentiated type, whereas a reddish or isochromatic lesion is endoscopically diagnosed as a differentiated type

The diagnostic algorithm of WLE used to differentiate undifferentiated‐type from differentiated‐type EGC was based on the color of the lesions (Figure 1).^16^, ^17^ A lesion paler than the surrounding mucosa was diagnosed as an undifferentiated type.

### Evaluation with M‐NBI

**FIGURE 2:**
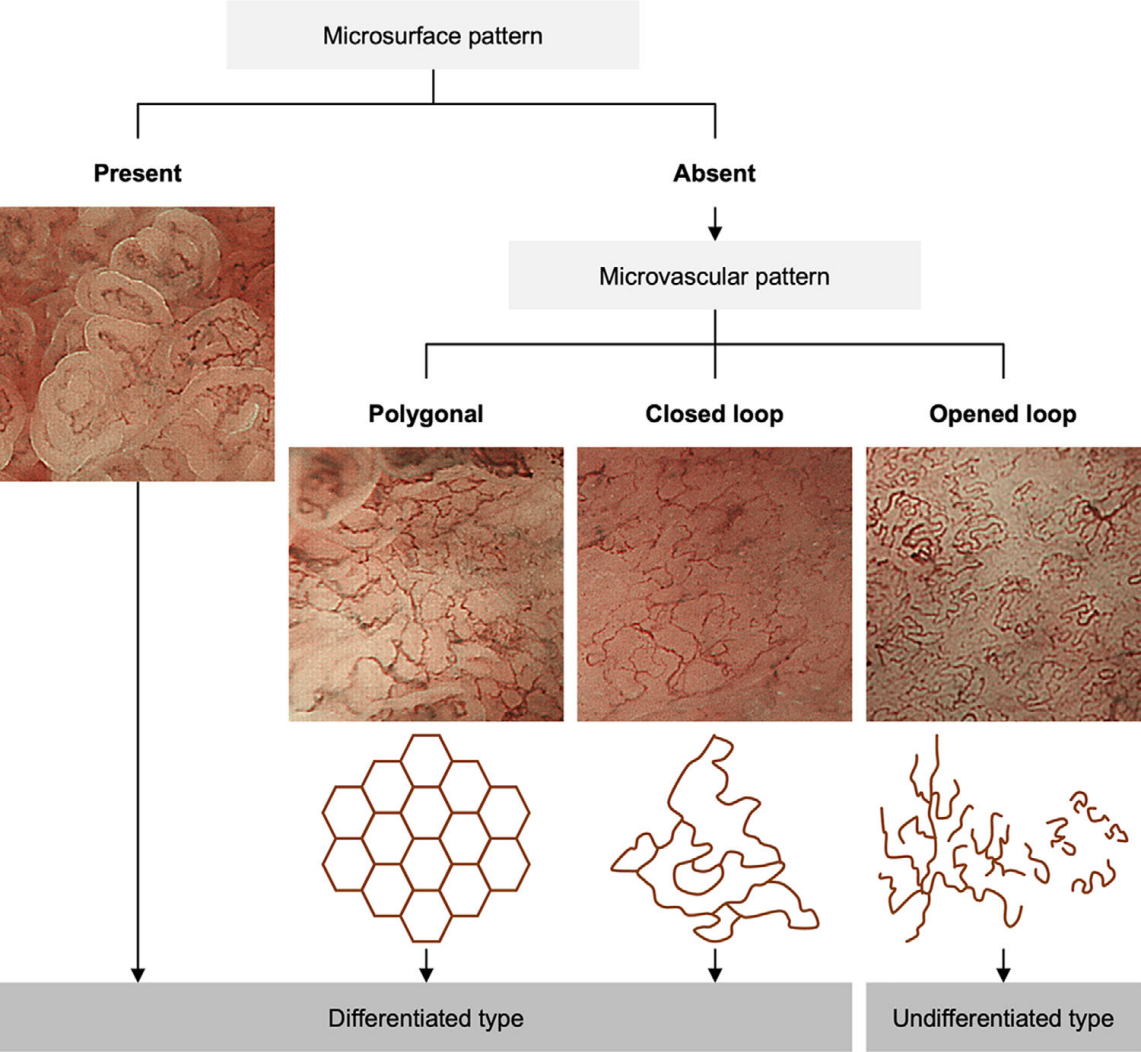
Diagnostic algorithm of magnifying narrow‐band imaging for differentiating undifferentiated‐type from differentiated‐type gastric cancer. The lesion with a microsurface pattern is endoscopically diagnosed as a differentiated type. If the lesion does not have a microsurface pattern, the microvascular pattern is evaluated. Polygonal or closed‐loop type is endoscopically diagnosed as a differentiated type, whereas opened‐loop type is endoscopically diagnosed as an undifferentiated type

The diagnostic algorithm of M‐NBI used to distinguish undifferentiated‐type from differentiated‐type EGC was based on previous reports (Figure 2).^18^, ^19^, ^20^, ^21^ Lesions with absent microsurface pattern and opened‐loop microvascular pattern, i.e., undifferentiated‐type pattern, were diagnosed as undifferentiated‐type.

### Histopathological diagnosis

All biopsy and resected specimens were histologically evaluated using hematoxylin and eosin staining. The pathologists were blinded to the endoscopic diagnosis for histological type. The histological diagnosis of EGC was made in accordance with the revised Vienna Classification.^22^ In this trial, categories 4 (noninvasive, high‐grade neoplasia) and 5 (invasive neoplasia) were classified as cancer, while categories 1 (negative for neoplasia), 2 (indefinite for neoplasia), and 3 (noninvasive, low‐grade neoplasia) were classified as non‐cancer. The histological type of EGC was diagnosed in accordance with the Japanese Classification of Gastric Carcinoma.^2^, ^14^ Well‐ and moderately‐differentiated tubular adenocarcinoma and papillary adenocarcinoma were classified as differentiated type, and poorly differentiated adenocarcinoma and signet‐ring cell carcinoma were classified as undifferentiated type. Mucinous adenocarcinoma was classified as differentiated or undifferentiated type in each case based on the degree of glandular differentiation. Mixed type histology of differentiated and undifferentiated types in a biopsy specimen was regarded as undifferentiated type.

### Outcomes

The primary and key secondary endpoints were on‐site diagnostic accuracy and specificity to distinguish undifferentiated‐type from differentiated‐type EGC, respectively. The reason for defining specificity as a key secondary endpoint was because avoidance of misdiagnosis of differentiated‐type as undifferentiated‐type may reduce over‐surgery for lesions ≥2 cm or lesions with an ulcer scar. The sensitivity, positive likelihood ratio, and negative likelihood ratio for distinguishing undifferentiated‐type from differentiated‐type EGC were secondary endpoints. In order to achieve a one‐to‐one correspondence between endoscopic and histological findings, the histological diagnosis of a biopsy specimen obtained from the center of the lesion was used for the reference standard in the main analysis. As a subset analysis, the diagnostic performance of M‐NBI for undifferentiated‐type EGC, according to the lesion color, was evaluated. In addition, diagnostic performance based on the dominant subtypes of resected specimens, which is also clinically relevant, was calculated as a sensitivity analysis. All adverse events were evaluated in accordance with the Common Toxicity Criteria for Adverse Events 4.03.

### Statistical analysis

Sample sizes were calculated to compare primary and key secondary endpoints between WLE and M‐NBI. In a pilot study using the aforementioned algorithms,^23^ 7.1% (4/56) of EGCs were misdiagnosed by M‐NBI, despite being correctly diagnosed by WLE, and 21.4% (12/56) were misdiagnosed by WLE, despite being correctly diagnosed by M‐NBI. Using McNemar's test with a two‐sided α of 0.05 and power of 0.8, 117 lesions were required to compare accuracy (for the primary endpoint). It was found that 9.8% (4/41) of differentiated‐type EGCs were misdiagnosed by M‐NBI, despite being correctly diagnosed by WLE, and 24.4% (10/41) were misdiagnosed by WLE, despite being correctly diagnosed by M‐NBI.^23^ Using McNemar's test with a two‐sided α of 0.05 and power of 0.8, 132 differentiated‐type EGCs were required to compare accuracy. Assuming that the proportion of the differentiated type among EGCs was similar to that in a recent multicenter clinical trial (80.8%, 277/343),^24^ 163 lesions were required to compare specificity for the undifferentiated type (for the key secondary endpoint). To assess not only the primary endpoint but also the key secondary endpoint, 163 lesions were required. Finally, the total sample size was set to 207 cases, considering 10% of excluded cases and 16.2% of the false‐positive rate of biopsy diagnosis for cancer.^25^

Baseline characteristics were summarized as a median and range for continuous variables and as a proportion for categorical variables. The diagnostic performances of WLE and M‐NBI were assessed by accuracy, sensitivity, specificity, and likelihood ratio, and they were described with a 95% confidence interval. McNemar's test was used to compare the diagnostic performance. A *p*‐value <0.05 was considered to indicate statistical significance. All statistical analyses were conducted using R software, version 3.6.3 (R Foundation for Statistical Computing, Vienna, Austria; http://cran.r‐project.org/).

## RESULTS

### Patient enrollment and background

**FIGURE 3:**
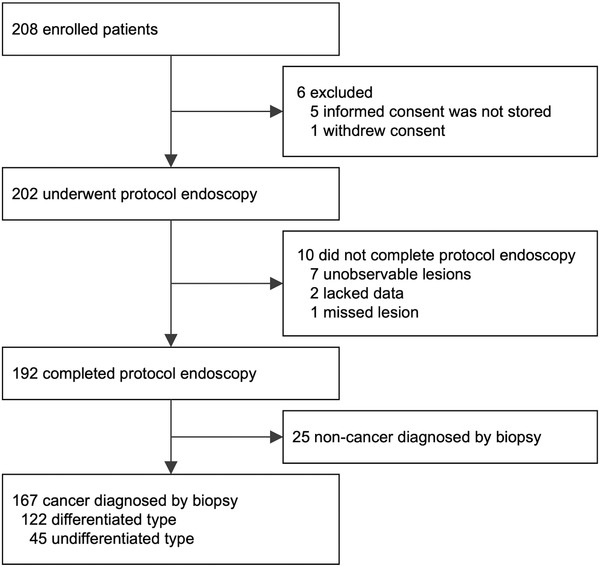
Patient flowchart

Between September 2018 and September 2019, 208 patients were enrolled from six tertiary care institutions in Japan. The consent forms of five patients were not stored, and one patient withdrew consent after enrollment. Among 202 patients who underwent protocol endoscopic examination, the diagnostic procedure and biopsy were completed in 192 patients by 41 participating endoscopists. The median number of biopsy specimens was 1 (range, 1–2 specimens) per lesion. In 25 patients, histological diagnosis of the biopsy specimen was made as non‐cancer. Finally, 167 lesions (122 differentiated‐type and 45 undifferentiated‐type EGCs) were included in the main analysis (Figure 3). Patient characteristics are shown in Table 1.

**TABLE 1:** Demographics of the study subjects

Clinicopathological characteristic	n = 167
Median age (years, range)	69 (34–93)
Sex	
Male	115 (69)
Female	52 (31)
*Helicobacter pylori* status	
Current infection	63 (38)
Past infection	93 (56)
Non‐infection	11 (7)
Endoscopy	
GIF‐Q240Z	5 (3)
GIF‐H260Z	27 (16)
GIF‐FQ260Z	4 (2)
GIF‐H290Z	131 (78)
Lesion location	
Upper third	33 (20)
Middle third	69 (41)
Lower third	65 (39)
Macroscopic type	
Depressed (0‐IIc/0‐IIc + III)	137 (82)
Flat (0‐IIb)	12 (7)
Mixed (others)	18 (10)
Endoscopic diameter (mm)	20 (5−100)
Histological type	
Differentiated‐type	122 (73)
Undifferentiated‐type	45 (27)

Data are presented as median (range) or *n* (%).

### Diagnostic performance to distinguish the histological types of gastric cancer

The accuracy, sensitivity, and specificity for undifferentiated‐type EGC were 80% (73%–86%), 69% (53%–82%), and 84% (77%–90%) with WLE, and 82% (75%–88%), 53% (38%–68%), and 93% (87%–97%) with M‐NBI, respectively (Table 2). Specificity was significantly higher with M‐NBI than with WLE (*p* = 0.041), but there was no significant difference in accuracy and sensitivity between WLE and M‐NBI (*p* = 0.755 and 0.190, respectively). Diagnostic performance of M‐NBI for undifferentiated‐type gastric cancer according to the lesion color is presented in Table 3.

**TABLE 2:** Diagnostic performance of white‐light endoscopy (WLE) and magnifying narrow‐band imaging (M‐NBI) for undifferentiated‐type gastric cancer

**Method**	**Accuracy, % (95% CI)**	**Sensitivity, % (95% CI)**	**Specificity, % (95% CI)**	**PLR (95% CI)**	**NLR (95% CI)**
WLE	80 (73–86)	69 (53–82)	84 (77–90)	4.4 (2.8–7.0)	0.37 (0.24–0.57)
M‐NBI	82 (75–88)	53 (38–68)	93 (87–97)	7.2 (3.6–14.4)	0.50 (0.36–0.69)
*p*‐value	0.755	0.190	0.041		

Abbreviations: CI, confidence interval; NLR, negative likelihood ratio; PLR, positive likelihood ratio.

**TABLE 3:** Diagnostic performance of magnifying narrow‐band imaging (M‐NBI) for undifferentiated‐type gastric cancer according to the lesion color

**Lesion color**	**Accuracy, % (95% CI)**	**Sensitivity, % (95% CI)**	**Specificity, % (95% CI)**
Reddish or isochromatic *n* = 117	90 (83–95)	50 (23–77)	95 (89–98)
Pale *n* = 50	64 (49–77)	55 (36–73)	79 (54–94)

Abbreviation: CI, confidence interval.

Of the 192 patients who completed the protocol endoscopic examination, 145 patients received ER and 40 underwent surgery. After exclusion of two special‐type EGCs and three non‐cancerous lesions, finally, 180 lesions were diagnosed as common‐type cancer (135 differentiated‐type and 45 undifferentiated‐type) in the resected specimens. The accuracy, sensitivity, and specificity for undifferentiated‐type EGC in reference to the dominant subtypes of resected specimens were 81% (75%–87%), 71% (56%–84%), and 84% (77%–90%) in WLE, and 84% (78%–89%), 56% (40%–70%), and 94% (89%–97%) in M‐NBI, respectively (Table 4). Specificity was significantly higher with M‐NBI than with WLE (*p* = 0.019), but there was no significant difference in accuracy and specificity between WLE and M‐NBI (*p* = 0.451 and 0.146, respectively).

**TABLE 4:** Diagnostic performance for undifferentiated‐type dominant gastric cancer in the resected specimen

**Modality**	**Accuracy, % (95% CI)**	**Sensitivity, % (95% CI)**	**Specificity, % (95% CI)**	**PLR (95% CI)**	**NLR (95% CI)**
WLE	81 (75–87)	71 (56–84)	84 (77–90)	4.6 (3.0–7.1)	0.34 (0.22–0.54)
M‐NBI	84 (78–89)	56 (40–70)	94 (89–97)	9.4 (4.6–19.3)	0.47 (0.34–0.66)
*p*‐value	0.451	0.146	0.019		

Abbreviations: CI, confidence interval; M‐NBI, magnifying narrow‐band imaging; NLR, negative likelihood ratio; PLR, positive likelihood ratio; WLE, white‐light endoscopy.

### Adverse events

No ≥Grade 2 adverse event occurred in any of the 202 patients.

## DISCUSSION

In this multicenter prospective study, we did not find a difference in the overall accuracy between M‐NBI and WLE for diagnosis of histological type of EGC. Currently, the Japanese guideline for endoscopic diagnosis of EGC states that diagnosis of histological type of EGC should be made comprehensively by endoscopic finding and histological finding of biopsy specimens^26^. However, the level of evidence for the statement is very weak. This study result must increase evidence level in this aspect.

**FIGURE 4:**
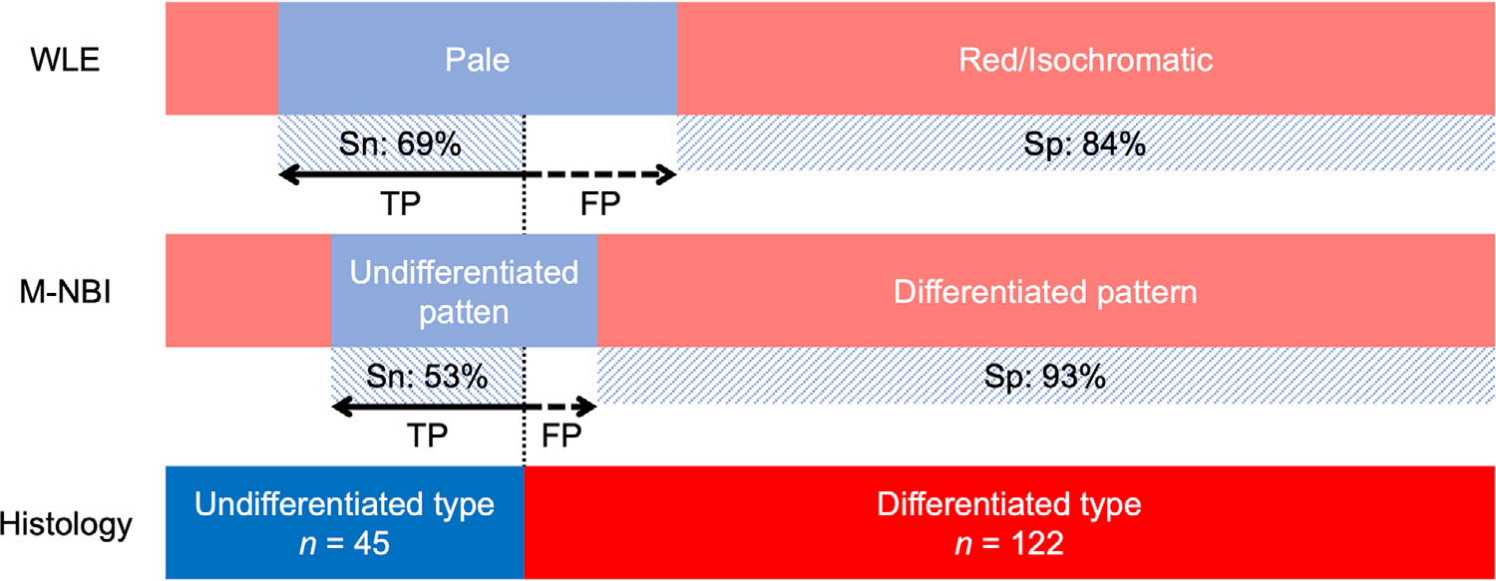
True‐positive and false‐positive rates in each examination. FP, false‐positive; M‐NBI, magnifying narrow‐band imaging; Sn, sensitivity; Sp, specificity; TP, true‐positive; WLE, white‐light endoscopy

M‐NBI showed higher specificity but lower sensitivity for the diagnosis of undifferentiated‐type EGCs. Most undifferentiated‐type EGCs appeared pale in WLE, showing 69% of sensitivity for undifferentiated‐type EGC, and a part of differentiated‐type EGC appeared pale (specificity of 84%, Figure 4). Meanwhile, the undifferentiated‐type pattern in M‐NBI decreased the false‐positive rate of WLE for diagnosis of the undifferentiated‐type EGC, but it also decreased the true‐positive rate (sensitivity). In subset analysis based on the lesion color, M‐NBI showed 79% specificity for undifferentiated‐type EGC for the pale lesions in WLE. If a differentiated‐type EGC is misdiagnosed as an undifferentiated‐type, gastrectomy may be indicated for the lesion and the patient loses the opportunity for treatment via ER. Using M‐NBI in addition to WLE enables a more accurate diagnosis and avoidance of over‐surgery in such cases. In contrast, if an undifferentiated‐type EGC is misdiagnosed as a differentiated type, ER may be indicated for the lesion. However, such cases can be treated by additional surgery after a histological diagnosis of the resected specimens. We did the sub‐analyses in addition to the lesion color, but we could not find any trends for each subset (Table S1).

We speculated two reasons for the low sensitivity of M‐NBI in this study. First, in our preliminary study, M‐NBI diagnosis by an expert endoscopist improved both sensitivity and specificity for the histological type of gastric cancers.^23^ When the expert endoscopist reviewed endoscopic images, the opened‐loop microvascular pattern was underdiagnosed in several cases. Evaluation of microvessels in M‐NBI needs certain experiences, therefore we suspect that further training of endoscopists or use of computer‐aided diagnosis may improve sensitivity.^27^, ^28^ Second, undifferentiated‐type EGC often exists subepithelially underneath the non‐neoplastic foveolar epithelium,^29^ therefore, such lesions were misdiagnosed as differentiated‐type EGC because of the presence of microsurface pattern of covering non‐neoplastic epithelium.

The principle to discriminate undifferentiated‐type from differentiated‐type EGC in WLE and NBI is different. An undifferentiated‐type EGC looks pale in WLE because of a reduction in hemoglobin content.^17^ Distinguishing the histological type of EGC by M‐NBI is based on differences in microsurface structure and microvascular architecture. A key histological feature of differentiated‐type EGC is ductal formation. For differentiated‐type EGC, marginal crypt epithelium (microsurface structure) of the cancerous duct is visible in M‐NBI.^30^ Otherwise, in case the cancerous ducts are too narrow or shallow, the marginal crypt epithelium is invisible (absent),^31^ and only network‐shaped microvessels (polygonal/closed‐loop microvessels) that surround cancerous ducts are seen.^18^ On the other hand, for undifferentiated‐type EGC, the marginal crypt epithelium is absent and non‐network shaped microvessels (opened‐loop microvessels) are seen, owing to the absence of the ductal formation.

Among 31 published articles for histological type diagnosis of EGC by M‐NBI, there were three prospective studies.^19^, ^21^, ^32^ Two single‐center studies evaluated the characteristic findings of the histological type of EGC using M‐NBI, but there were no comparative data by WLE.^19^, ^21^ A multicenter comparative study showed no significant difference between WLE and M‐NBI for diagnosis of histological type of EGCs: the accuracies 96.4% and 96.8%, and the sensitivity for the differentiated‐type, which correspond to specificities for the undifferentiated‐type, were 99.0% and 99.5%.^32^ However, the proportion of the undifferentiated‐type EGC among the study subjects was quite low (7%) and most lesions were small and had no ulceration because only cases of ER were included in that study. We suspect that such selection bias in the study subjects might increase the diagnostic performance of both WLE and M‐NBI in that study. Diagnosis of histological type is important for all EGCs to determine the indication of ER. A strength of our study is that patients undergoing both ER and/or surgery were included.

Our study had limitations. First, we did not design this study as a randomized controlled trial. Although the endoscopic finding of each method was evaluated independently, the diagnostic value of M‐NBI contained carrying over effect from WLE diagnosis and the comparison between WLE and M‐NBI diagnoses was indirect. In clinical practice, M‐NBI diagnosis is always performed subsequently to WLE, therefore we considered that making a study arm using only M‐NBI diagnosis without WLE was impractical. The prospectively obtained data in this study reflected the diagnostic performance of both methods in real clinical settings. Second, only patients with EGC were recruited, therefore the usefulness of M‐NBI for advanced gastric cancers was unknown. However, diagnosis of histological type is the most important in EGC among cancers in all T‐stages because it is related to the indication of ER. Third, we defined biopsy diagnosis as a reference standard instead of the diagnosis of the resected specimen in this study. As a result, 25 patients were excluded from the main analysis because the biopsy specimen taken in the protocol examination was diagnosed as non‐cancer. When we reviewed these misdiagnosed specimens, most lesions were underdiagnosed because of low‐grade atypia of the neoplastic glands. Otherwise, there was no neoplastic gland suggesting sampling error by forceps biopsy. Thus, biopsy diagnosis for EGC has a risk of misdiagnosis.^25^ However, we considered biopsy to the exact endoscopic observation point was the most feasible method to achieve a one‐to‐one correspondence between an endoscopic finding and histology. When the histopathological examination of the resected specimen was used as a reference standard, a precise corresponding evaluation was difficult, especially for surgical specimens. We also evaluated the diagnostic performance of M‐NBI for the dominant histological type of resected specimens, and the results were similar (Tables 2 and 4). Fourth, the only color was included in the diagnostic algorithm of WLE, because the color had been demonstrated as the most useful predictor for the histological type of depressed or flat‐type EGCs in previous studies.^16^, ^17^ Moreover, although *H. pylori* eradication is reported to alter the color of the background mucosa,^33^ we did not find a significant difference in the diagnostic accuracy of WLE based on the *H. pylori* status (Table S1), thus its influence may be small.

In conclusion, this prospective study demonstrated that additional use of M‐NBI did not improve the overall accuracy of WLE for diagnosis of non‐ulcerated flat or depressed type undifferentiated EGCs. However, it improved the specificity of WLE and may reduce surgical over‐treatment by preventing misdiagnosis of differentiated‐type EGC as undifferentiated‐type.

## CONFLICT OF INTEREST

The authors declare that they have no conflict of interest.

## FUNDING INFORMATION

This study was funded by The Yasuda Medical Foundation.

## Supporting information


**Supplementary Table**. Subset analyses of diagnostic performance of WLE and M‐NBI for undifferentiated‐type gastric cancer
