# Supplementary material for: Diagnosis of histological type of early gastric cancer by magnifying narrow‐band imaging: A multicenter prospective study
Source: DEN Open. 2021 Sep 28;2(1):e61. doi: 10.1002/deo2.61 (PMC8828242; doi:10.1002/deo2.61)
Supplement: Supplementary file 1 — Supplementary Table. Subset analyses of diagnostic performance of WLE and M‐NBI for undifferentiated‐type gastric cancer [file DEO2-2-e61-s001.docx]

**Supplementary Table.** Subset analyses of diagnostic performance of WLE and M-NBI for undifferentiated-type gastric cancer

| **Clinical subsets** | | **n** | **Methods** | **Accuracy, %**  **(95% CI)** | **Sensitivity, %**  **(95% CI)** | **Specificity, %**  **(95% CI)** |
| --- | --- | --- | --- | --- | --- | --- |
| *H. pylori* status | Current infection | 63 | WLE | 75  (62–85) | 68  (43–87) | 77  (62–89) |
|  |  |  | M-NBI | 79  (67–89) | 63  (38–84) | 86  (73–95) |
|  | Past infection | 93 | WLE | 84  (75–91) | 71  (48–89) | 88  (78–94) |
|  |  |  | M-NBI | 84  (75–91) | 43  (22–66) | 96  (88–99) |
|  | Non-infection | 11 | WLE | 82  (48–98) | 60  (15–95) | 100  (42–100) |
|  |  |  | M-NBI | 82  (48–98) | 60  (15–95) | 100  (42–100) |
| Tumor location | Upper third | 33 | WLE | 73  (55–87) | 67  (22–96) | 74  (54–89) |
|  |  |  | M-NBI | 79  (61–91) | 50  (12–88) | 85  (66–96) |
|  | Middle third | 69 | WLE | 83  (72–91) | 78  (56–93) | 85  (71–94) |
|  |  |  | M-NBI | 80  (68–88) | 57  (35–77) | 91  (79–98) |
|  | Lower third | 65 | WLE | 82  (70–90) | 56  (30–80) | 90  (78–97) |
|  |  |  | M-NBI | 86  (75–94) | 50  (25–75) | 98  (89–100) |
| Tumor size | ≤20 mm | 111 | WLE | 84  (76–90) | 65  (43–84) | 89  (80–94) |
|  |  |  | M-NBI | 88  (81–94) | 57  (35–77) | 97  (90–99) |
|  | >21 mm | 56 | WLE | 73  (60–84) | 73  (50–89) | 74  (56–87) |
|  |  |  | M-NBI | 70  (56–81) | 50  (28–72) | 82  (66–93) |
| Tumor depth | M | 117 | WLE | 83  (75–89) | 71  (51–87) | 87  (78–93) |
|  |  |  | M-NBI | 88  (81–93) | 50  (31–69) | 100  (94–100) |
|  | SM or deeper | 45 | WLE | 73  (58–85) | 65  (38–86) | 79  (59–92) |
|  |  |  | M-NBI | 67  (51–80) | 59  (33–82) | 71  (51–87) |
|  | CI, confidence interval; M-NBI, magnifying narrow-band imaging; WLE, white-light endoscopy. | | | | | |
